# Educational training to improve opioid overdose response among health center staff: a quality improvement initiative

**DOI:** 10.1186/s12954-023-00803-z

**Published:** 2023-06-30

**Authors:** Audrey Stephenson, Alessandra Calvo-Friedman, Lisa Altshuler, Sondra Zabar, Kathleen Hanley

**Affiliations:** 1 NYC Health + Hospitals/Gotham Health, Gouverneur, 227 Madison St., New York, NY 10002 USA; 2 Present Address: West Chester University of Pennsylvania, West Chester, PA USA; 3 Department of Medicine, NYU Grossman School of Medicine, New York, NY USA

**Keywords:** Quality improvement, Opioid overdose, Opioid use disorder, Stigma, Educational intervention

## Abstract

**Background:**

There were seven opioid overdoses in this New York City (NYC) federally qualified health center from December 2018 through February 2019, reflecting the rising rate of overdose deaths in NYC overall at the time. In response to these overdoses, we sought to increase the readiness of health center staff to recognize and respond to opioid overdoses and decrease stigmatizing attitudes around opioid use disorder (OUD).

**Methods:**

An hour-long training focusing on opioid overdose response was administered to clinical and non-clinical staff of all levels at the health center. This training included didactic education on topics such as the overdose epidemic, stigma around OUD, and opioid overdose response, as well as discussion. A structured assessment was administered immediately before and following the training to evaluate change in knowledge and attitudes. Additionally, participants completed a feedback survey immediately after the training to assess acceptability. Paired *t*-tests and analysis of variance tests were used to assess changes in pre- and post-test scores.

**Results:**

Over 76% of the health center staff participated in the training (*N* = 310). There were large and significant increases in mean knowledge and attitudinal scores from pre- to post-test (*p* < .001 and *p* < .001, respectively). While there was no significant effect of profession on attitudinal change scores, profession did have a significant effect on knowledge change scores, with administrative staff, non-clinical support staff, other healthcare staff, and therapists learning significantly more than providers (*p* < .001). The training had high acceptability among participants from diverse departments and levels.

**Conclusions:**

An interactive educational training increased staff’s knowledge and readiness to respond to an overdose as well as improved attitudes toward individuals living with OUD. *Trial registration:* This project was undertaken as a quality improvement initiative at the health center and as such was not formally supervised by the Institutional Review Board per their policies. Further, per the guidelines of the International Committee of Medical Journal Editors, registration is not necessary for clinical trials whose sole purpose is to assess an intervention’s effect on providers.

**Supplementary Information:**

The online version contains supplementary material available at 10.1186/s12954-023-00803-z.

## Background

Rates of overdose deaths involving heroin and synthetic opioids other than methadone have increased every year from 2010 to 2019 in New York City (NYC) [1, 2]. Illicitly manufactured fentanyl emerged in the national drug supply around 2014, greatly contributing to the increase in opioid overdose deaths nationally and in NYC specifically [3]. Alarmingly, there were seven opioid overdoses in this NYC health center from December 2018 through February 2019. The majority of these overdoses occurred in the first-floor public restrooms, a setting which is not unusual given that public and hospital restrooms are a common place for people who inject drugs to use [4]. Multiple physicians at the health center who responded to the overdoses reported that they did not feel adequately prepared. Additionally, some overdoses were not recognized quickly, with one victim not receiving the appropriate care until approximately 30 min after he overdosed.

In response to the national overdose crisis, overdose response educational initiatives have been implemented in various settings with promising results. For example, an intervention on overdose education and naloxone administration implemented in an academic health system showed that such an educational intervention is feasible in a healthcare setting [5]. Additionally, it has been shown that only a five-to-ten-minute educational session on naloxone administration is sufficient to have a significant improvement in opioid users’ overdose response [6]. Further, naloxone distribution and overdose education programs for bystanders have been shown to be effective at increasing knowledge of overdose response and rates of overdose recovery [7] as well as reducing deaths from opioid overdose [8]. Overall, evidence suggests that overdose education and naloxone distribution programs lead to long-term increases in knowledge around overdose response, improve attitudes around opioid overdose and naloxone, adequately prepare participants to respond to overdoses, and decrease overdose mortality [9].

In addition to lack of public education on overdose response, stigma, which has been recognized as a fundamental cause of health inequalities [10], is also associated with increased risk for individuals living with OUD. This is reflected in research finding that many health professionals have negative perceptions of individuals living with substance use disorders (SUDs) [11, 12], resulting in the patient receiving suboptimal care [11], delaying seeking treatment, deemphasizing their pain, and not informing their provider of their substance use [13]. Further, it has been found that people who use drugs’ perceived stigma is associated with having a non-fatal overdose, suggesting that stigma increases risk of an overdose [14].

Educational interventions have been found to be effective at reducing stigma around individuals with SUDs. One study found that an intervention involving education, discussion, and experiential de-escalation training using videos and role-play improved police officers’ self-efficacy when interacting with individuals with cocaine and alcohol dependence as well as decreased their desired social distance from them [15]. Another study found that a training involving education regarding the effect of drugs and alcohol on pregnancy, a tutorial on how to treat a pregnant patient with SUD, and a simulated patient encounter increased second-year medical students’ comfort levels when treating pregnant patients with SUD [16]. Further, research has also found that education on the role of childhood trauma in development of an SUD reduces stigma around the disorder [17] and that SUD terminology has an effect on the orator’s stigma, whether they are aware of it or not [18, 19]. Additionally, a sensitization training for healthcare professionals in five South African provinces involving education to counter commonly-held negative attitudes and societal stigma around individuals living with SUD resulted in reduced perception of illegal drug use as immoral as well as increased comfort in treating this population [20].

Given the evidence suggesting that educational trainings focused on overdose response and stigma reduction have been effective at reducing overdose mortality, two primary care staff at the health center, a primary care physician and an AmeriCorps Member serving in the addiction medicine clinic, decided to implement a similar didactic program to improve health center staff’s response to the overdoses in the facility and reduce stigmatizing attitudes around OUD. The purpose of this study is to evaluate the effect of this training series on staff knowledge and attitudes surrounding overdose response and OUD.

## Methods

### Setting

The intervention was carried out at Gouverneur Health, a health center within the NYC Health + Hospitals network, which is located in the Lower East Side of Manhattan in New York City. It is also a federally qualified health center, providing a safety net to vulnerable individuals including those who are uninsured or experiencing homelessness. The patients serviced in this facility have a diversity of racial and ethnic backgrounds and speak a variety of languages. The facility houses both a skilled nursing facility as well as comprehensive outpatient care services, including an addiction medicine clinic which was recently established at the time of this intervention. The initiative was only implemented on the outpatient unit portion of the facility and had full support from the facility’s administration.

### Intervention

The standard protocol in this health center is that when a medical emergency such as an opioid overdose occurs, the rapid response team is notified and urgently responds to the incident. The team attends to the overdose victim’s medical needs, administers naloxone, and ensures that they are connected with ongoing medical attention. Clinical staff rotate through the rapid response team such that different staff are on duty on different days.

In order to increase staff’s preparedness to respond to overdoses, two investigators, a primary care physician and an AmeriCorps Member who coordinated the addiction medicine clinic’s overdose response training and SUD clinical service, spearheaded this quality improvement initiative aimed at improving staff’s knowledge of and attitudes around opioid overdose response and OUD. 29 trainings were conducted at the health center and its two satellite clinics from April to July 2019. The training participants (*N* = 310) included clinical as well as non-clinical staff of all levels at the health center from physicians to clerical staff and hospital police officers. Clinical as well as non-clinical staff were included as staff of various positions were first to encounter overdoses in the facility. Department leadership were notified of the training by email, and they then coordinated scheduling with the health center administrative leadership. Staff were required to attend and, in total, over 76% of the health center staff were given the training. Training size was limited to 26 participants. Staff in each department were grouped together for trainings and for larger departments, such as primary care, the professions were each given their own training (e.g., primary care nurses). Hospital police were trained first as they were often the first on the scene of an overdose and were thus a priority group.

The two investigators who spearheaded the initiative developed the training through a combination of their prior knowledge as well as additional research. They first created an outline based on their general knowledge about SUDs, the overdose crisis, stigma around OUD, treatments for OUD, and opioid overdose response, and then conducted additional research to provide greater detail to the presentation content (e.g., incorporating quantitative data on the overdose crisis in NYC). The AmeriCorps Member investigator contributed material to the presentation based on her knowledge as a certified naloxone dispenser in New York State, and some content of the presentation was also adapted from the NYC Department of Health and Mental Hygiene’s training of naloxone dispensers presentation. While the overall content of each presentation was the same for all training groups, slight adjustments were made according to participants’ levels of medical training. The interactive training included information on (1) the overdose crisis in the surrounding city; (2) the prevalence and lethality of fentanyl; (3) how the overdose crisis affected the health center; (4) some causes of OUD, including adverse childhood experiences; 4) stigma around OUD, including compassionate language; (5) treatments for OUD; and (6) overdose response, including an interactive experience with a naloxone nasal spray. The intervention was conducted by the AmeriCorps Member investigator who was certified as a naloxone trainer and dispenser through an opioid overdose prevention program at the health center. Naloxone kits were provided by the New York State Department of Health and distributed to all interested participants free of charge. In total, approximately 177 naloxone kits were given out. After the training, participants asked questions and completed a feedback survey. The entire presentation lasted one hour.

There were periodic meetings among the authors throughout the intervention which resulted in the presenter’s enhanced presentation skills, increased discussion among the participants, and increased clarity of the presentation. The fundamental content of the training was not changed. The presenter also continuously incorporated participants’ feedback to refine the presentation and enhance her presenting skills.

### Measures

Identical anonymous pre- and post-tests lasting about five minutes were administered immediately before and following the training. Following the post-test, participants also completed a feedback survey on their experience during the presentation.

#### Demographics

The pre- and post-tests inquired about personal demographics including gender, profession, age, number of years employed at the health center, and department, all of which were voluntary to disclose. Of these demographics, only data on department was collected in the first training as a meeting among the investigators resulted in the addition of the other demographic items beginning in the second training. As there were only four participants in the first training, this adjustment to data collection did not significantly contribute to the amount of missing data. Data on race and ethnicity was not collected in order to maintain anonymity among participants from small departments. At the nineteenth training, feedback surveys were linked to each participant’s anonymous ID.

#### Pre- and post-tests

##### Opioid and overdose-related knowledge (7 multiple choice items, 3 true/false items)

The pre-and post-tests contained various items assessing knowledge of and preparedness to respond to an overdose (i.e., the “Knowledge” section; see Additional file 1). The first six items from the Knowledge section were adapted from the validated Opioid Overdose Knowledge Scale [21] and assessed signs and symptoms of an overdose, steps in responding to an overdose, and how to use naloxone. The next four items were developed by the first and second authors and assessed knowledge of compassionate language, withdrawal symptoms, and treatments for OUD. The items adapted from the Opioid Overdose Knowledge Scale as well as one of the newly-created items were all multiple choice, each with four options plus a “Don’t know” option. The remaining three newly-created items were in a True/False/“Don’t know” format. Two responses for the item assessing compassionate language were considered correct, and items that were left unanswered or had multiple responses were considered incorrect. Overall, the items on the Knowledge section yielded a possible total score from 0 to 10. The Knowledge section had strong internal reliability (pre-test Cronbach’s *α* = 0.78, post-test Cronbach’s *α* = 0.71).

##### Opioid and overdose-related attitudes (6 Likert-type items)

The tests also contained various items assessing attitudes around overdose response and interacting with patients with OUD (i.e., the Attitudinal section; see Additional file 1). The first three items in the Attitudinal section were adapted from the validated Opioid Overdose Attitudes Scale [21] and assessed preparedness and comfortability in responding to an overdose. The next two items were adapted from the validated Medical Condition Regard Scale [22] and assessed comfortability when interacting with patients with OUD and compassion toward this population. The final item in this section was created by the first and second authors and assessed beliefs around an individual with OUD’s ability to regain a stable life. Notably, the last three items of the Attitudinal section assess stigmatizing attitudes around individuals with OUD. All of the Attitudinal section items were on a 5-point Likert-type scale (Not at all, Slightly, Moderately, Very, Extremely) scored 1–5, respectively. When an item had multiple responses, the mean was used in analyses. The scores for all items in this section were averaged to get a total attitudinal score. One of the items in the Attitudinal section (“How panicked would you be if you saw an overdose?”) did not cluster well with the others so it was excluded from analyses. Without this item, the Attitudinal section had strong internal reliability (pre-test Cronbach’s *α* = 0.82, post-test Cronbach’s *α* = 0.91).

#### Acceptability (10 Likert-type items, 4 free response items)

To assess acceptability, participants completed a feedback survey consisting of 10 Likert-type items and four free response prompts. The first nine items were scored on a 5-point Likert-type scale (Not at all, Slightly, Moderately, Very, Extremely) scored 1–5, respectively, and assessed the instructor’s and presentation’s quality. The tenth item had a 5-point Likert-type scale (Poor, Fair, Good, Very good, Excellent) format, scored 1–5, respectively, and evaluated the overall quality of the presentation. The four free response prompts inquired about the presentation’s strengths, areas for improvement, and discussion time, and provided a space for additional comments (see Additional file 2).

### Data analysis

Paired *t*-tests were used to assess the change in Knowledge and Attitudinal scores from pre- to post-test. A Shapiro–Wilk test found that knowledge scores had a significant departure from normality for the pre- (*W*(289) = 0.96, *p* < 0.001) and post-test (*W*(289) = 0.72, *p* < 0.001). An *F* test found that the Knowledge pre- and post-test scores did not have homogenous variances, *F*(1, 289) = 2.696, *p* < 0.001, but it was determined that a *t-*test was still appropriate for the data given the large and equal sample size (*N* = 290) [23]. Similarly, a Shapiro–Wilk test found that attitudinal scores also had a significant departure from normality for the pre- (*W*(289) = 0.98, *p* < 0.001) and post-test (*W*(289) = 0.97, *p* < 0.001). Additionally, an *F* test found that the scores did not have significantly different variances, *F*(1, 289) = 1.035, *p* = 0.77. Despite the non-normal distributions, it was determined that a *t*-test was still appropriate for the data given the large and equal sample size (*N* = 290) [23], the Shapiro–Wilk test’s sensitivity to deviations from normality [24], and because the data appeared fairly normal upon visual inspection. We computed Cohen’s *d* effect size on each of the two *t*-scores to assess the size of the difference.

“Change scores” were created by subtracting the pre- from post-test scores in the Knowledge and Attitudinal sections such that a higher change score indicated greater improvement. These change scores were used to analyze whether profession was a mediating variable in the intervention’s effect on Knowledge and Attitudinal scores. Analysis of variance (ANOVA) tests were used to assess the effect of profession on knowledge and attitudinal change scores. A Shapiro–Wilk test found that knowledge scores for providers, administrative staff, and other healthcare staff were not normally distributed, all of which were skewed right. However, it was determined that an ANOVA was still appropriate due to the robustness of the test [25]. Additionally, a Bartlett’s test found that the groups’ Knowledge change scores did not have equal variance, *b*(5) = 12.34, *p* < 0.05. Tukey HSD tests were used to find which professions’ scores were statistically different after the ANOVA. A Shapiro–Wilk test found that attitudinal change scores for providers, other healthcare staff, and non-clinical support staff were not normally distributed, rather all were right skewed. Despite this, it was determined that an ANOVA was still appropriate due to the robustness of the test [25]. Additionally, a Bartlett’s test found that the groups’ attitudinal change scores had significantly different variances, *b*(5) = 23.84, *p* < 0.001. All analyses were computed using R Studio Version 4.3.0 [26].

Given that the investigators made efforts to improve the presentation throughout the intervention, a linear regression was performed to assess whether training number had an effect on knowledge and attitudinal change scores.

To analyze feedback scores, we used the mean of the Likert-type items as the summary score for acceptability. To analyze the free response feedback, we used an inductive approach to identify themes in the data as we did not have a priori expectations of the responses. One coder analyzed the data for themes, and the research team reviewed the results.

## Results

### Missing data

Of the 310 total participants, 16 did not complete either the pre- or post-test, one completed the post-test during the training, and three did not complete any items in the Attitudinal section. Thus, only 290 participants were eligible for analyses. Of these participants, 31 did not disclose their profession, yielding 259 participants eligible for analysis by profession.

### Demographic and background characteristics

Of the 290 participants who were included in analysis, 204 (70.3%) identified as female, and 24 (8.3%) did not disclose their gender identity. The mean age was 42.5 (range = 22–69), and 123 (42.4%) did not disclose their age. The mean years worked at the health center was 9.4 (mode = > 1), and 38 (13.1%) did not disclose their length of employment. 31 (10.7%) declined to indicate their profession. See Table 1 for participant characteristics. To compare the different professions, we grouped similar professions in our analyses based on level of education and clinical training. Group size ranged from 19 to 53. See Table 2 for the groups of professions.

**Table 1:** Participant characteristics

Variable	Characteristics	Frequency (*N* = 290)	Percentage (%)
Age	18–29	24	8.3
	30–39	56	19.3
	40–49	29	10.0
	50–59	39	13.4
	60–69	18	6.2
	Declined to disclose	124	42.8
Gender	Male	62	21.4
	Female	204	70.3
	Declined to disclose	24	8.3
Years at the health center*	0–4	107	36.9
	5–9	39	13.4
	10–14	37	12.8
	15–19	24	8.3
	20–24	19	6.6
	25–29	17	5.9
	30–34	5	1.7
	35–39	3	1.0
	40–44	0	0.0
	45–49	1	0.3
	Declined to disclose	38	13.1

*For participants’ responses regarding number of years working at the health center, some participants only provided their minimum number of years (e.g., “5+”). For those participants, we assigned them to the lowest value indicated (e.g., “5+” was included in the 5–9 year group). We decided to include this data in recognition that those who have worked at the health center for a greater number of years were less likely to report the exact number, and we wanted to minimize the likelihood of skewing the data toward those who had worked at the health center for less time

**Table 2:** Groups of participants’ professions

Group	Profession	Frequency (*N* = 290)	Percentage (%)
Providers	Physician, Pharmacist, Optometrist, Nurse Practitioner, Physicians’ Assistant	53	18.3
Nurses	RN, Head Nurse, Staff Nurse, Assistant Director of Nursing	29	10.0
Therapists	Psychologist, Psychologist in Training, Social Worker, Peer Counselor	35	12.1
Non-clinical support staff	Assistant System Analyst, Clerical Associate/Clerk, Community Liaison, Client Navigator, Secretary, Hospital Police, Special Officer, Watch Person, Institutional Aide, Floor Tech, Housekeeping Aide	52	17.9
Administrative	Administrator, CEO, Director, Manager/Assistant Coordinating Manager, Supervisor, Accountable Care Manager, Administrator on Duty	19	6.6
Other healthcare	Patient Care Associates, Respiratory Therapist, Phlebotomist, Dietician, Public Health Advisor, Dental Assistant, Dental Hygienist, Pharmacy Tech, Lab Tech, Eye and Vision Technician, Technician, Lab Supervisor, Lab Director	71	24.5
None	Declined to disclose	31	10.7

### Change in OUD and overdose-related knowledge

A paired *t*-test found a large (Cohen’s *d* = 1.52) and significant increase in mean knowledge scores from pre- (*M* = 5.16, *SD* = 2.74) to post-test (*M* = 8.80, *SD* = 1.67), *t*(289) = 26.10,* p* < 0.001, CI.95 = 3.37–3.92, two-tailed test. Separate paired *t*-tests also found a significant increase in knowledge scores from pre- to post-test for each profession (see Table 3 for means and *p*-values). A one-way between groups ANOVA found a significant effect of profession on the Knowledge change scores, *F*(5, 253) = 5.67, *p* < 0.001. A post hoc Tukey HSD test indicated significant differences in the mean change scores for the providers (*M* = 2.32, *SD* = 2.16) compared with administrative staff (*M* = 4.21, *SD* = 2.51), non-clinical support staff (*M* = 4.40, *SD* = 2.75), other healthcare staff (*M* = 4.11, *SD* = 2.40), and therapists (*M* = 3.80, *SD* = 1.62). There was no significant difference between the change scores for the other groups.

**Table 3:** Mean pre- and post-test scores by profession for the knowledge section

Group	Mean pre-test score	Mean post-test score	*P*-value*
Providers	7.53	9.85	> .001
Nurses	6.28	9.48	> .001
Other healthcare	4.11	8.23	> .001
Therapists	5.54	9.34	> .001
Administrative	5.32	9.53	> .001
Non-clinical support staff	3.06	7.46	> .001
Did not disclose	5.45	8.90	

*Following a Bonferroni correction, we used an α of .0083

### Change in OUD and overdose response-related attitudes

A paired *t*-test found a large (Cohen’s *d* = 0.81) and significant increase in mean Attitudinal scores from pre- (*M* = 2.63, *SD* = 0.91) to post-test (*M* = 3.36, *SD* = 0.89), *t*(289) = 17.62, *p* < 0.001, CI.95 = 0.65–0.81, two-tailed test. Separate paired *t*-tests also found a significant increase in attitudinal scores from pre- to post-test for each profession (see Table 4 for means and *p*-values). A one-way between groups ANOVA did not find a significant effect of profession on the attitudinal change scores, *F*(5, 253) = 0.880, *p* = 0.50.

**Table 4:** Mean pre- and post-test scores by profession for the attitudinal section

Group	Mean pre-test score	Mean post-test score	*P*-value*
Providers	3.18	3.79	> .001
Nurses	2.92	3.50	> .001
Other healthcare	2.43	3.15	> .001
Therapists	2.71	3.53	> .001
Administrative	2.88	3.66	> .001
Non-clinical support staff	2.17	3.01	> .001
Did not disclose	2.43	3.18	

*Following a Bonferroni correction, we used an α of .0083

### Change over time

A linear regression found that the effect of training number on change scores was nonsignificant for both the knowledge change scores (*F*(1, 288) = 0.03, *p* = 0.854) and the attitudinal change scores (*F*(1, 288) = 0.75, *p* = 0.387).

### Participant evaluation of acceptability

The scores on the Likert-type items of the feedback survey were high (*M* = 4.52, min = 4.43, max = 4.61). Participants’ responses on the free response items provided insight into which parts of the training they enjoyed as well as suggestions for improvement. Among perceived strengths of the presentation, predominant themes included clarity, level of detail, organization, and relevance of the content. Some components of the presentation which the participants found strengthened the training were the presentation slides, the discussion of overdose response, and the video of a simulated response to an overdose. Additionally, participants reported that specific characteristics of the presenter enhanced the presentation, such as that she was knowledgeable and open to questions.

Regarding perceived areas for improvement of the presentation, themes included making the presentation more interactive, providing handouts of key points, making the training longer, incorporating case studies, and incorporating a video of response to an overdose. When multiple participants made the same suggestion, the presenter tried to modify the presentation accordingly. For example, after two participants in the first three trainings suggested incorporating a video, the presenter made this modification and in subsequent trainings six participants reported that the video enhanced the presentation. Additionally, the instructor started distributing handouts of key points in the last two trainings after two participants suggested this change.

Answers to the free response item regarding discussion time were overall positive, with 85 out of the 93 participants who responded indicating that the training had the right amount of discussion. Common themes among the free response item for additional comments included general praise for the presentation, notes that the presentation was informative, statements that it should be given to more people, and comments on the importance of the content. Participants from several departments and levels reported that they found the presentation generally positive (see Additional file 3). See Table 5 for the feedback themes, frequencies, and exemplars.

**Table 5:** Free-response results of the feedback survey

Themes	Frequencies	Exemplars
*Strengths—overall presentation*		
Clear	34	Knowable presentation, Simple and direct, Concise, “Well-articulated in a language that is easy to understand”
Detailed	18	Detailed, Informative, Well-explained
Organized	12	Well-structured, Organized
Relevant information	8	Pertinent information, Practical, Relevant, Useful
Engaging	6	Pre/posttest was engaging, The use of visual aids and the presenter made the training interactive, All participants were engaged, Interactive
Good scheduling	3	Short, On time, Quick
*Strengths—components of the presentation*		
Slides	13	PowerPoint was helpful, Slides were effective
Overdose response	7	Actionable guidance on responding to an overdose, Narcan training, Very useful information on how to administer naloxone
Video	5	Great video
Discussion	3	Discussion, Q&A
Data	3	The real life number on this issue, Statistics
*Strengths—the presenter*		
Knowledgeable	4	Presenter was well-informed, Presenter has good command of subject matter
Presentation style	4	Friendly presenter, The presenter made the training interactive, Presenter seemed passionate about the topic, Calmness and consistency of delivery
Openness to questions	3	Good with questions, I appreciated the open manner in which she took and responded to questions
*Suggestions for improvement*		
More interactive	7	Could allow for more discussion, Maybe have more participants answer questions before presenting answers, More interactive, Implementing role play
More extensive overdose response and naloxone administration demonstration	7	Video of interactions and Narcan administration, Visual of an actual OD, “Test kits” to allow us to feel what it is like to use naloxone, Review using Narcan kit a bit more, If we were taught CPR, Specific steps for linking people with SUD to care
Handouts	3	Maybe a handout with key points
Longer	3	Could be a bit longer, especially the part about the effects of naloxone
Case studies	3	Scenarios, Examples and case study
*Amount of discussion time*		
Too much	0	
Too little	2	
Right amount	85	
Response didn’t address the question	6	
*Additional comments*		
General praise	15	Excellent job, Wonderful presentation
Informative	5	Informative, Keep up the informative work
Should be given to more people	5	We should do more so everyone can be prepared, Keep doing this for as many people as possible
The importance and utility of the topic	5	Needs to be in every hospital because the information is much needed, I know there are many people living with this disorder and it’s great to be able to know what to do in any situation
Praise for the presenter	3	The presenter was comfortable and aware of OUD, The presenters did a good job

## Discussion

In this initiative, we explored the efficacy and acceptability of an hour-long educational training to improve OUD and overdose response-related knowledge and attitudes at a health center. Participants’ Knowledge scores improved over the course of the intervention, indicating that the training increased participants’ knowledge of opioid overdose response, use of compassionate language, and treatment for OUD. Participants’ Attitudinal scores also improved, indicating that the intervention improved participants’ attitudes around OUD. Further, given that the last three attitudinal items assess participants’ stigma around individuals with OUD (see Additional file 1), the improvement in Attitudinal scores may indicate a stigma reduction among participants as well. Not surprisingly, we found that administrative staff, non-clinical support staff, other healthcare staff, and therapists’ Knowledge change scores improved more than those of providers, indicating that they learned more from the intervention. This was likely because the providers had the highest base rate knowledge, evidenced by their left-skewed pre-test scores. This finding highlights the importance of including non-clinical staff of all levels in interventions targeting knowledge around OUD and opioid overdose at health centers. Additionally, profession did not have an effect on the attitudinal change scores, indicating that all staff’s stigmatizing attitudes around OUD decreased equally. Participants from diverse departments and levels gave high scores and positive free response answers on the feedback survey, indicating that the training was successfully adapted to each group.

This quality improvement initiative contributed to the field in a few ways. First, it was distinct from several others of its kind in that it aimed to train all clinical and non-clinical health center employees in recognition that not only could any staff member encounter an overdose, but all staff play an important role in caring for patients with OUD. Additionally, participants of diverse departments and levels found the presentation highly acceptable, indicating that such a training can be successfully adapted to diverse groups. This training was also especially impactful since it was conducted in a high-risk area. In 2021, the rate of opioid overdose deaths in New York was 25.2 per 100,000, higher than the national rate of 24.4 per 100,000 [27]. Future research should continue to implement and study similar interventions in areas with the highest need since that is the population where these changes will have the greatest impact. Other future research should also explore ways of making interventions shorter. This intervention was time-intensive to implement because it was an addition to standard protocols instead of a modification of existing protocols and because of the hour-long duration of each training. It was likely only feasible because there was an AmeriCorps Member in the clinic with the time available to dedicate to the project, and other settings may have difficulty implementing similar initiatives if the implementation team does not have the time availability. Future studies should explore and refine ways to make the intervention increasingly more time-efficient. Additionally, the intervention may have been more effective at improving participants’ attitudes around OUD if the presentation had used a contact-based approach to reduce stigma, such as if someone with lived experience were to lead the training. Research suggests that educational interventions are more effective at reducing stigma around a substance-using population if paired with a contact-based approach [28–30]. Thus, future interventions should incorporate contact-based approaches, whether through use of a video or the assistance of someone with lived experience.

There are a few limitations to this initiative. First, the long-term effects of this initiative are unknown as follow-up data was not collected, and thus future interventions should assess follow-up data. Second, attitudes were self-reported and thus subject to inaccuracy. For instance, 75 participants gave the same response for all questions in the “Attitudinal” section of the pre- and/or post-test. Specifically, 16 participants gave the same response on the pre-test, 43 gave the same response on the post-test, and 16 gave the same response on both. Because almost three times as many participants gave uniform responses on the post- compared with the pre-test, this uniformity could suggest that participants were less thoughtful when completing the post-test due to being eager to leave after the training. Future research could address this shortcoming by assessing implicit as well as explicit attitudes, as has been suggested previously [31]. Finally, the lack of a control group weakens the initiative’s internal validity; the improved Attitudinal scores could have been because the participants felt they were expected to become less biased over the course of the training. Thus, future research should also include control groups in similar interventions.

## Conclusions

The number of opioid overdoses has risen dramatically across the country in recent years [32]. Given this increase, efforts to train healthcare professionals to respond to opioid overdoses as well as reduce their stigmatizing attitudes around this disorder are more important than ever. Our intervention found that an hour-long interactive educational training significantly improved health center staff’s OUD and overdose response-related knowledge and attitudes, with non-clinical staff evidencing particularly large knowledge gains. Additionally, the training was well-received by participants.

### Supplementary Information

**Additional file 1**. Pre- and post-test.

**Additional file 2**. Feedback survey.

**Additional file 3**. Data.

## Data Availability

With the exception of a few pieces of data, all other data generated or analyzed during this study are included in this published article and its supplementary information files. The excluded data (participant identification number, gender, age, and number of years working at the health center) are not publicly available in order to maintain participants’ anonymity.

## Abbreviations

ANOVA: Analysis of variance
NYC: New York City
OUD: Opioid use disorder
SUD: Substance use disorder
