# Correction: Educational training to improve opioid overdose response among health center staff: a quality improvement initiative

**DOI:** 10.1186/s12954-023-00843-5

**Published:** 2023-08-02

**Authors:** Audrey Stephenson, Alessandra Calvo‑Friedman, Lisa Altshuler, Sondra Zabar, Kathleen Hanley

**Affiliations:** 1 NYC Health + Hospitals/Gotham Health, Gouverneur, 227 Madison St., New York, NY 10002 USA; 2 Present Address: West Chester University of Pennsylvania, West Chester, PA USA; 3 Department of Medicine, NYU Grossman School of Medicine, New York, NY USA

**Correction : Harm Reduction Journal (2023) 20:83** 10.1186/s12954-023-00803-z

Following publication of the original article [1], in this article the affiliation of all the authors has been updated and the same will read as follows:

^1^NYC Health + Hospitals/Gotham Health, Gouverneur, 227 Madison St., New York, NY 10002, USA.

^2^Present Address: West Chester University of Pennsylvania, West Chester, PA, USA.

^3^Department of Medicine, NYU Grossman School of Medicine, New York, NY, USA.

The original article has been corrected.

