# Supplementary material for: Educational training to improve opioid overdose response among health center staff: a quality improvement initiative
Source: Harm Reduct J. 2023 Jun 30;20:83. doi: 10.1186/s12954-023-00803-z (PMC10311901; doi:10.1186/s12954-023-00803-z)
Supplement: Supplementary file 1 — Additional file 1. Pre- and post-test. [file 12954_2023_803_MOESM1_ESM.docx]

**Pre- and Post-Test**

The following is the pre-/post-test that participants completed immediately before and following the intervention.

Last 4 digits of your phone number: Date:

Improving Opioid Overdose Response: Pre/Post-Test

Gender: Position: Age:

Years working at health center: Department:

**Multiple Choice (Circle the correct answer for each question)**

1. Which of the following is NOT one of the signs and symptoms of an opioid overdose?
   1. Slow breathing or not breathing
   2. Agitated behavior
   3. Blue or pale nails or lips
   4. Unresponsive
   5. Don’t know
2. Which of the following is NOT one of the steps in responding to an opioid overdose?
   1. Giving rescue breaths if the person is not breathing
   2. Giving stimulants (e.g. cocaine or black coffee)
   3. Placing the person in the recovery position after breathing is resumed
   4. Administering naloxone
   5. Don’t know
3. What is naloxone used for?
   1. To reverse an opioid overdose
   2. To reverse an amphetamine overdose
   3. To reverse a cocaine overdose
   4. To reverse any overdose
   5. Don’t know
4. How long does naloxone take to start having an effect?
   1. 2-8 minutes
   2. 8-10 minutes
   3. 11-20 minutes
   4. 21-40 minutes
   5. Don’t know
5. How long do the effects of naloxone last?
   1. Less than 20 minutes
   2. 30-90 minutes
   3. 2-6 hours
   4. 7-12 hours
   5. Don’t know
6. Which of the following statements is NOT true about responding to an opioid overdose?
   1. If the first dose of naloxone has no effect a second dose should be given after two minutes.
   2. To check for responsiveness, the victim should be shaken roughly.
   3. After the naloxone wears off, the person could begin to overdose again if they do not receive medical attention.
   4. Naloxone can provoke withdrawal symptoms.
   5. Don’t know
7. Which of the following is an example of compassionate language?
   1. Substitution therapy
   2. Clean
   3. Recovery
   4. Person living with opioid addiction/opioid use disorder
   5. Don’t know

**True, false, or don’t know? (Circle the correct answer) DK = Don’t know**

1. Medication for addiction treatment makes the life of a person living True/False/DK

with OUD (opioid use disorder/opioid addiction) more stable.

1. People living with OUD need to use opioids to keep from feeling sick. True/False/DK
2. There are three FDA-approved treatments for OUD. True/False/DK

**Personal Opinion: Mark your answer to each question.**

|  | Not at all | Slightly | Moderately | Very | Extremely |
| --- | --- | --- | --- | --- | --- |
| How prepared are you to respond to an opioid overdose? |  |  |  |  |  |
| How comfortable are you administering naloxone on your own? |  |  |  |  |  |
| How panicked would you be if you saw an overdose? |  |  |  |  |  |
| How comfortable are you with interacting with patients with OUD? |  |  |  |  |  |
| How compassionate do you feel toward patients with OUD? |  |  |  |  |  |
| How capable do you think that patients with OUD are at regaining stable lives? |  |  |  |  |  |
