# Supplementary material for: Educational training to improve opioid overdose response among health center staff: a quality improvement initiative
Source: Harm Reduct J. 2023 Jun 30;20:83. doi: 10.1186/s12954-023-00803-z (PMC10311901; doi:10.1186/s12954-023-00803-z)
Supplement: Supplementary file 2 — Additional file 2. Feedback survey. [file 12954_2023_803_MOESM2_ESM.docx]

**Feedback Survey**

The following is the feedback survey that participants completed immediately following the intervention.

Feedback Questions

**The Instructors:**

|  | Not at all | Slightly | Moderately | Very | Extremely |
| --- | --- | --- | --- | --- | --- |
| Did the instructors answer questions effectively? | 1 | 2 | 3 | 4 | 5 |
| Did the instructors present in a clear and structured manner? | 1 | 2 | 3 | 4 | 5 |
| Did the instructors seem knowledgeable about the subject? | 1 | 2 | 3 | 4 | 5 |
| Were the instructors prepared? | 1 | 2 | 3 | 4 | 5 |
| Were the instructors engaging? | 1 | 2 | 3 | 4 | 5 |

**The Presentation:**

|  | Not at all | Slightly | Moderately | Very | Extremely |
| --- | --- | --- | --- | --- | --- |
| Was the presentation well-organized? | 1 | 2 | 3 | 4 | 5 |
| Did the information presented seem relevant to you? | 1 | 2 | 3 | 4 | 5 |
| Were the graphics helpful? | 1 | 2 | 3 | 4 | 5 |
| Did you enjoy the presentation? | 1 | 2 | 3 | 4 | 5 |

| Overall, I would rate this presentation as: | Poor | Fair | Good | Very Good | Excellent |
| --- | --- | --- | --- | --- | --- |

**Additional Feedback:**

What were the strengths of this presentation?

How can this presentation be improved?

Did the instructors allow too much, too little, or the right amount of time for discussion?

Additional comments:

***Thank you for your feedback!***
